# A likely association between low mannan-binding lectin level and brain fog onset in long COVID patients

**DOI:** 10.3389/fimmu.2023.1191083

**Published:** 2023-06-16

**Authors:** Roberta Bulla, Lucrezia Rossi, Giovanni Furlanis, Chiara Agostinis, Miriam Toffoli, Andrea Balduit, Alessandro Mangogna, Marco Liccari, Giorgia Morosini, Uday Kishore, Paolo Manganotti

**Affiliations:** ^1^ Department of Life Sciences, University of Trieste, Trieste, Italy; ^2^ Neurology Unit, Department of Medical, Surgical and Health Sciences, Cattinara University Hospital, ASUGI, University of Trieste, Trieste, Italy; ^3^ Institute for Maternal and Child Health, IRCCS Burlo Garofolo, Trieste, Italy; ^4^ Department of Medical, Surgical and Health Science, University of Trieste, Trieste, Italy; ^5^ Department of Veterinary Medicine, United Arab Emirates University, Al Ain, United Arab Emirates

**Keywords:** mannan-binding lectin (MBL), SARS-CoV-2, brain fog, long covid, complement system

## Abstract

Brain fog can be described as a constellation of new-onset neuropsychiatric sequelae in the post-acute phase of COVID-19 (long COVID). The symptoms include inattention, short-term memory loss, and reduced mental acuity, which may undermine cognition, concentration, and sleep. This cognitive impairment, persisting for weeks or months after the acute phase of SARS-CoV-2 infection, can significantly impact on daily activities and the quality of life. An important role for the complement system (C) in the pathogenesis of COVID-19 has emerged since the beginning of pandemic outbreak. A number of pathophysiological characteristics including microangiopathy and myocarditis have been attributed to dysregulated C activation due to SARS-CoV-2 infection. Mannan-binding lectin (MBL), the first recognition subcomponent of the C lectin pathway, has been shown to bind to glycosylated SARS-CoV-2 spike protein, genetic variants of *MBL2* are suggested to have an association with severe COVID-19 manifestations requiring hospitalization. In the present study, we evaluated MBL activity (lectin pathway activation) and levels in the sera of a cohort of COVID-19 patients, presenting brain fog or only hyposmia/hypogeusia as persistent symptoms, and compared them with healthy volunteers. We found significantly lower levels of MBL and lectin pathway activity in the sera of patients experiencing brain fog as compared to recovered COVID-19 patients without brain fog. Our data indicate that long COVID-associated brain fog can be listed among the variegate manifestations of increased susceptibility to infections and diseases contributed by MBL deficiency.

## 1 Introduction

Acute and chronic manifestations due to severe acute respiratory syndrome coronavirus 2 (SARS-CoV-2) infection have continued to stimulate an ever-growing body of research. Much has been already published about the acute phase symptoms (*i.e.*, anorexia, myalgia, productive sputum, fever, exhaustion, dyspnoea) of Coronavirus Disease 2019 (COVID-19), as initial non-specific upper airway symptoms due to viral infections before developing into pneumonia (1–3). From the neurological point of view, manifestations of the central and peripheral nervous systems, such as meningitis, encephalitis, myelitis, Guillain-Barré syndrome, Miller Fisher syndrome and cerebrovascular diseases, have been reported (4–10). In particular, recent scientific literature has revealed anosmia and dysgeusia as typical symptoms of the acute phase, which persist for weeks or months after infection and can be included among the so-called “long COVID symptoms” (11, 12). Prominent among the long-term consequences of COVID-19 are neuropsychiatric sequelae, also referred to as “brain fog” (13). The brain fog summarizes a range of variegate neurological complications, including inattention, short-term memory loss and reduced mental acuity (14). Impaired cognition, attention, concentration, and sleep are the commonly reported neuropsychiatric manifestations associated with the post-recovery phase of COVID-19 (14). This cognitive impairment may last for weeks or even months after the acute phase. To understand the pathophysiology of the brain fog, clinicians are currently trying to identify structural, metabolic and perfusion alterations in the brain using advanced neuroimaging techniques (15–19). Many ideas have been proposed to explain the aetiology of brain fog-associated symptoms, such as SARS-CoV-2 neuro-invasion, abnormal systemic and neuroimmunological response, cytokine overactivation, neuroglial dysfunction, virus-induced coagulopathy and endotheliopathy; however, the exact pathogenic mechanisms remain largely unknown (13, 20–23).

The important role of the complement system (C) in the pathogenesis of COVID-19 has emerged since the beginning of the COVID-19 (24–26). In general, C1q, a major player in the complement-mediated neuroinflammation (27, 28), can be produced by microglia cells; in concert with TNF-α and IL-1α, it can induce the polarization through a A1 phenotype, which is the neurotoxic and pro-inflammatory phenotype of astrocytes (29, 30). Reactive astrocytes increase the expression level of many genes of the classic pathway, such as C1r, C1s, C3, and C4, which are harmful for the neurovascular unit (28). However, little is known about the of role of the C system in neuroinflammation due to long COVID.

A recent study demonstrated that the subcomponent of the C lectin pathway, mannan-binding lectin (MBL), can bind to glycosylated SARS-CoV-2 spike protein, activating the C, and inhibiting SARS-CoV-2 cell entry *in vitro* (31). Single-nucleotide polymorphisms (SNPs) of *MBL2* impact serum levels and functional activity of MBL (32). Interestingly, Stravalaci et al. demonstrated that genetic polymorphisms at the *MBL2* locus are associated with COVID-19 severity (31). Therefore, MBL may act as a double-edged sword in the resistance to infection as well as in the pathogenesis of COVID-19. MBL deficiency is the most frequent C immunodeficiency, occurring in more than 10% of general population (33, 34). Interestingly, MBL polymorphisms have been reported as predisposing factors for susceptibly to infectious as well as systemic diseases such as systemic lupus erythematosus, rheumatoid arthritis and sepsis (33, 35–37).

Since the pathogenesis of the neurological “long COVID syndrome” remains largely unclear (20) and the C cascade is known to be involved in brain development, homeostasis, injury and regeneration (38), the aim of the study was to evaluate a correlation between the neurological “long COVID syndrome” and the C pathway. In particular, we determined MBL levels and activity in the sera of a cohort of COVID-19 patients affected by the “long COVID syndrome”. Thus, we divided the cohort into patients who developed brain fog and those who complained of hyposmia/hypogeusia as persistent symptoms, comparing them with healthy volunteers.

## 2 Materials and methods

### 2.1 Participants

The study cohort comprised patients who were referred to the Neuro-Long-COVID ambulatory service of the University Hospital of “Cattinara” (Trieste, Italy) between 1^st^ November 2021 and 1^st^ March 2022, and were selected from a previously described cohort (39). Participants were screened for the presence of self-reported neurological symptoms experienced during the post-acute COVID-19 period (SARS-CoV-2 positivity was determined by nasopharyngeal swab and RT-qPCR). Neurological symptoms had to be persistent or occuring *ex novo* at least after 4 weeks from acute COVID-19 manifestations. Among participants, 32 subjects reported symptoms of brain-fog (BF +ve group); 13 subjects complained of only hyposmia/hypogeusia as persistent symptoms (BF -ve group). Three volunteers without persistent neurological symptoms, but with history of SARS-CoV-2 positivity, were also included in the BF -ve group. Eighteen healthy controls (CTRL group) without a history of positive SARS-CoV-2 nasopharyngeal swab were also recruited. A total of 66 blood samples were collected, processed and randomly selected for experiments. Anamnestic data of all participants were collected (**Table 1**) and an extensive neurological assessment of the BF +ve and BF -ve groups was performed. Cognitive screening test Montreal Cognitive Assessment (MoCA) was carried out on patients experiencing BF.

**Table 1:** Clinical features of the cohort.

Patients’ characteristics	BF +ve (n=32)	BF -ve (n =16)	CTRL (n= 18)
**Age** (years)	53.5 (44.75-58.25)	46.5 (31.75-62.25)	43.5 (28.5-53.75)
Sex			
Male (M)	8 (25%)	4 (25%)	5 (27.8%)
Female (F)	24 (75%)	12 (75%)	13 (72.2%)
**Time from Covid-19 testing (positive) and blood sampling** (days)	319.5 (289.8-356)	320 (279-368.8)	n.a.
Comorbidities			
Neurological	11 (34.4%)	3 (18.8%)	2 (11.1%)
Psychiatric	5 (15.6%)	0 (0%)	1 (5.6%)
Cardiovascular	11 (34.4%)	4 (25.0%)	4 (22.2%)
Respiratory	6 (18.8%)	1 (6.3%)	0 (0%)
Metabolic	10 (31.3%)	4 (25.0%)	2 (11.1%)
Malignancies	4 (12.5%)	0 (0%)	0 (0%)
Endocrine	6 (18.8%)	4 (25.0%)	3 (16.7%)
Rheumatologic	2 (6.3%)	1 (6.3%)	1 (5.6%)
Overweight/Obesity	8 (25.0%)	0 (0%)	4 (22.2%)
Acute Covid Symptoms			
Dyspnea	15 (46.9%)	1 (6.3%)	n.a.
Respiratory failure	2 (6.3%)	0 (0%)	n.a.
Use of non-invasive-ventilation (NIV)	1 (3.1%)	0 (0%)	n.a.
Long-Covid Symptoms			
Fatigue	12 (37.5%)	2 (12.5%)	n.a.
Dyspnea/Upper respiratory symptoms	12 (37.5%)	1 (6.3%)	n.a.
Myalgia/arthralgia	8 (25.0%)	0 (0%)	n.a.
Hyposmia/hypogeusia	5 (15.6%)	13 (81.3%)	n.a.
Insomnia	6 (18.8%)	0 (0%)	n.a.
Headache	4 (12.5%)	0 (0%)	n.a.
Mood disturbances	3 (9.4%)	0 (0%)	n.a.
Paresthesia	3 (9.4%)	0 (0%)	n.a.
Ocular problems	2 (6.3%)	0 (0%)	n.a.
Tinnitus	1 (3.1%)	0 (0%)	n.a.
Dizziness	1 (3.1%)	0 (0%)	n.a.
Fever (low grade)	1 (3.1%)	0 (0%)	n.a.
Tachycardia/palpitations	1 (3.1%)	0 (0%)	n.a.
GI tract symptoms	0 (0%)	0 (0%)	n.a.

Data are expressed as median (IQR: Q1-Q3) or total number (percentage, %). BF +ve, brain fog positive group; BF -ve, brain fog negative group; CTRL, control group; n.a., not applicable; NIV, non-invasive ventilation; GI, gastrointestinal.

General anamnestic data on all participants were collected for the presence of neurological, psychiatric, cardiovascular, respiratory, metabolic, endocrine and rheumatologic comorbidities, malignancies and overweight/obesity. Additionally, BF +ve and BF -ve patients were asked to provide information about the acute phase of SARS-CoV-2 infection, including the presence of acute upper respiratory symptoms, headache, myalgia or joint pain, hyposmia or anosmia, palpitations, diarrhoea or gastrointestinal tract symptoms, asthenia, dyspnoea, respiratory failure and the requirement of ventilation. Long COVID manifestations were extensively studied; following screening for symptoms lasting for more than four weeks after the infection onset; the patients were examined for persistent neurological symptoms (*i.e*., paraesthesia, hyposmia or anosmia, mood disturbances, insomnia, asthenia, headache, dizziness) and other persistent non-neurological symptoms (*i.e.*, myalgia or joint pain, ocular problems, tinnitus, persistent fever, palpitations, respiratory and gastrointestinal symptoms), besides cognitive impairment.

All clinical studies were performed according to the declaration of Helsinki. The protocol for this study was approved by the local ethics committee (CEUR-2021-OS-48).

### 2.2 Sample collection

During a follow-up visit, a 5-mL blood sample was collected by intravenous puncture using a vacuum collection system. The samples were transported, within 3 hours, to the Immunolaboratory of the University of Trieste, where they were processed to separate serum from blood cells. Serum was stored at -80°C until the time of analysis.

### 2.3 Quantitation of sVCAM-1, CRP and MBL levels

Commercial ELISA kits were used for the measurement of serum levels of soluble Vascular Cell Adhesion Molecule-1 (sVCAM-1; RayBiotech, Milan, Italy), C-reactive Protein (CRP; Invitrogen, ThermoFisher, Milan, Italy) and MBL (HyCult, Milan, Italy), following the manufacturer’s instructions. Absorbance was read using PowerWave X Microplate Reader (Bio-Tek Instruments) spectrophotometer.

### 2.4 Evaluation of complement pathways functionality

The activation of classical, alternative and lectin pathways was determined using a commercial kit Wieslab^®^ (Technogenetics, Milan, Italy). The assay was performed following the manufacturer’s instructions. The absorbance was read using PowerWave X Microplate Reader (Bio-Tek Instruments) spectrophotometer.

## 3 Results

### 3.1 Clinical characteristics of the patients

Demographic features, comorbidities, acute and long-COVID symptoms of the three groups (BF +ve, BF –ve and CTRL) are presented in **Table 1**. In particular, 32 patients from the Neuro-Long-COVID ambulatory service complaining of brain fog symptoms were enrolled in the brain fog group (BF +ve). Among these group, females were prevalent (75%) and the median age of the group was 53.5 years. Among the pre-existing comorbidities, the most common were neurological (34.4%), cardiovascular (34.4%), metabolic (31.3%) and overweight/obesity (25%). Neurological co-morbidities were represented by migraine, presence or history of a pituitary microadenoma, sciatic pain, and in one patient, history of episodes of subjective dizziness. None of the BF +ve patients had cognitive deficits before COVID-19. During the acute phase of SARS-CoV-2 infection, almost half of patients of the BF +ve group presented dyspnoea (46.9%), whereas only 2 individuals experienced respiratory failure and one subject required the use of non-invasive ventilation (NIV). Besides the cognitive deficits, the most frequently reported long COVID symptoms were fatigue, dyspnoea/upper respiratory symptoms and myalgia/arthralgia (37.5%, 37.5% and 25.0% of the total cases, respectively). Corrected median Montreal Cognitive Assessment (MoCA) score of this group was 24.5 (q1-q3 = 23.2-27.1).

As for the group without brain fog (BF -ve), a total of 16 cases were recruited from the Neuro-Long-COVID ambulatory service. The group had a high number of female patients (75%) and the median age was 46.5 years. The most frequent comorbidities were cardiovascular, metabolic and endocrine (25.0% each). Only one patient reported dyspnoea as a symptom of the acute phase of SARS-CoV-2 infection. Almost the entire group complained of hyposmia/hypogeusia as long COVID symptoms (81.3%).

With regard to healthy controls (CTRL), 18 people with median age of 43.5 years were selected (72.2% female volunteers). Most prevalent comorbidities were cardiovascular (22.2%), overweight/obesity (22.2%), and endocrine (16.7%) comorbidities.

### 3.2 Characterization of the inflammatory state of the patient cohort

**Figure 1:**
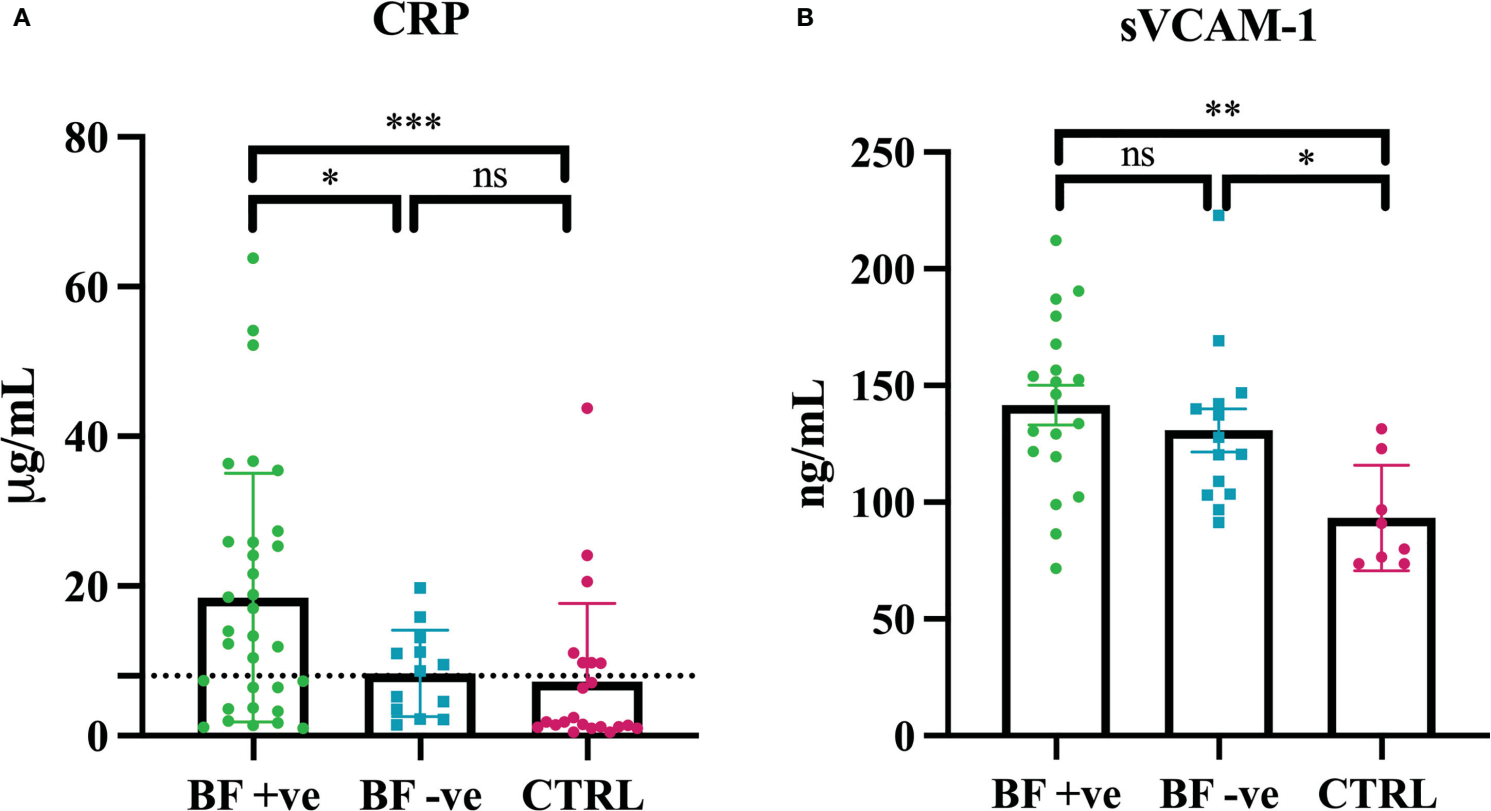
Serum levels of C-reactive protein (CRP) and soluble Vascular Cell Adhesion Molecule-1 (sVCAM-1). **(A)** CRP levels were measured in brain fog group (BF +ve; n = 32), experiencing only hyposmia/hypogeusia (BF -ve; n = 15) and in control non-infected patients (CTRL; n = 18). **(B)** sVCAM-1 levels were measured in brain fog group (BF +ve; n = 19), experiencing only hyposmia/hypogeusia (BF -ve; n = 14) and in control non-infected patients (CTRL; n = 8, control individuals were randomly selected). *p<0.05; **p<0.01; ***p<0.001; ns, not significant; T-test.

In order to characterize the inflammatory state and the endothelial dysfunction in our cohort of patients, we measured soluble Vascular Cell Adhesion Molecule-1 (sVCAM-1) and C-reactive protein (CRP) in their serum samples. Our results indicated the presence of significantly higher levels of CRP in BF +ve sera, as compared to both BF -ve and CTRL patients (**Figure 1A**). Furthermore, sVCAM-1 levels were higher in all the patients that had recovered for COVID-19 (BF +ve and BF -ve) compared to 8 patients from the CTRL group that were randomly chosen (**Figure 1B**).

### 3.3 Evaluation of MBL levels and complement pathway functionality in BF +ve and BF -ve patients

**Figure 2:**
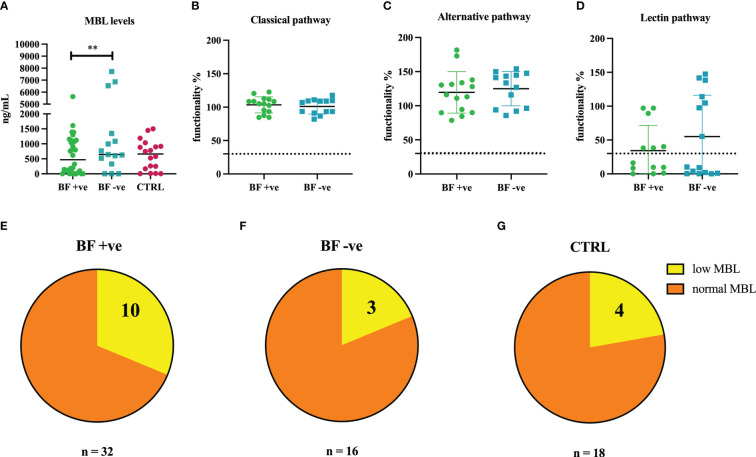
**(A)** Measurement of circulating levels of MBL in the sera of brain fog group (BF +ve, n = 32), experiencing only hyposmia/hypogeusia (BF -ve, n = 16) and non-infected control patients (CTRL, n = 18). **(B–D)** Evaluation of the functionality percentage of BF +ve and BF -ve patients using Wieslab ELISA kit. **(E–G)** Pie graphs representing the percentages of MBL low patients in BF +ve (31.3%), BF -ve (18.8%) and CTRL (22.2%) groups. **p<0.01; T-test.

MBL deficiency is the most frequent C deficiency worldwide (34). Based on the previous evidence of association between *MBL2* genetic polymorphisms and COVID-19 severity (31), we investigated a potential correlation between MBL deficiency, or low MBL levels, and the development of brain fog after COVID-19. We observed significantly lower levels of MBL in the sera of BF +ve patients compared to BF -ve group (**Figure 2A**).

Our results were confirmed by functional analysis of the three pathways of the C using Wieslab ELISA assay. Sera belonging to the BF +ve group presented a lower percentage of lectin pathway activation compared to BF -ve patients (**Figure 2D**), whereas no differences were found in the activation level of classical and alternative pathways (**Figures 2B, C**).

With a view to ascertain the distribution of patients with low or zero levels of MBL among the different groups, we noted that BF +ve group presented a higher percentage of MBL deficient subjects (31.3%; **Figure 2E**) as compared to BF -ve (18.8%; **Figure 2F**) as well as CTRL groups (22.2%; **Figure 2G**).

## 4 Discussion

The term long COVID refers to symptomatic manifestations persisting at least 12 weeks after SARS-CoV-2 infection (40, 41). It can reveal in both sexes and at all ages, despite being more frequently present in women and in older people (42). Many individuals infected with SARS-CoV-2 developed one or more long-lasting symptoms which strongly impair daily function and quality of life. Among the range of clinical manifestations associated with long COVID, neurological issues are the most frequent (7–9, 41), occurring approximatively in one third of long COVID patients (43–47). The most common neurological symptoms comprise fatigue, headache, attention disorder, cognitive impairment, ageusia, anosmia, memory loss, dizziness (41). Therefore, not only acute phase symptoms of COVID-19, but also post-COVID sequelae, is a matter of concern for the healthcare system as well as clinicians.

One of the most common concerns about long COVID is the frequent onset of the so called “brain fog”. Brain fog has been previously associated with viral aftermath (48), but also with chronic fatigue syndrome (49), chemotherapy treatments (50), fibromyalgia and other chronic conditions (51). Since brain fog is considered as one of the most debilitating post-COVID symptoms, understanding its pathophysiological mechanisms is an urgent clinical need.

Multiple explanations have been offered towards the aetiology of symptoms associated with brain fog, such as viral neuro-invasion, virus-induced coagulopathy and endotheliopathy, and abnormal immunological response (20). A recent study has implicated long-term tissue damage and unresolved inflammation due to viral persistence and lymphopenia as the main cause of long COVID (43). Interestingly, chronic inflammation, especially neuroinflammation, is frequently the driving force behind COVID-associated cognitive impairment (52). In order to assess the contribution of inflammation as well as endothelial dysfunction, we first measured the serum levels of C-reactive protein (CRP) and Vascular Cell Adhesion Molecule-1 (sVCAM-1), respectively. sVCAM-1 is considered a marker of chronic cerebral blood flow dysregulation due to cerebral endothelial damage (53), whereas CRP is an acute phase reactant, a well-known systemic marker for inflammation (54). Our data showed a significantly higher level of CRP in BF +ve sera compared to both BF -ve and CTRL groups. These results partially confirmed the observations of Mandal et al. who reported elevated levels of CRP in long COVID-19 patients (55). Thus, we specifically noted a chronic condition of inflammation with higer levels of inflammatory biomarkers in BF +ve patients. On the contrary, sVCAM-1 levels were higher in the patients who had recovered from COVID-19 (BF +ve and BF -ve) compared to the CTRL group, confirming that SARS-CoV-2 infection can be considered an endothelial disease.

The C system is a protective factor in the early stages of SARS-CoV-2 infection enhancing virus elimination; however, an excessive or aberrant activation of the C, leading to hyperinflammation and endothelial injury, has extensively been attributed to severe COVID-19 pathogenesis (56, 57). Interestingly, MBL may also function as a double-edged sword in COVID-19 (31). MBL is a key player in the innate immune defense system, which allows us to fight various pathogens by direct recognition and neutralization, acts both as an opsonin for pathogens as well as a recognition molecule in C activation via the lectin pathway. MBL is capable of binding human immunodeficiency virus-1 (HIV-1) and hepatitis virus B (HBV), contributing to host susceptibility to infection and disease progression (58, 59). Reduced levels of circulating MBL are associated with an increased risk of invasive pneumococcal disease, other bacterial infections (*e.g.*, *Staphylococcus aureus*, *Pseudomonas aeruginosa*, *Clostridium difficile)*, sepsis, and death from pneumonia (60–62).

Around 10-20% of humans harbour point mutations in the MBL gene (*MBL2*), which are associated with low MBL activity and consequent higher incidence of infections. In fact, polymorphisms in *MBL2* promoter and coding sequences adversely affect plasma levels, oligomeric state and ligand binding ability of MBL (63). Interestingly, genetic polymorphisms at the *MBL2* locus have previously been associated with susceptibility to SARS-CoV-2 infection (64), and more recently, with COVID-19 severity (31, 65). Due to its preponderant and ambivalent role in infection and pathogenesis, we aimed at investigating a potential contribution of MBL to long COVID, especially brain fog onset. Our study found significantly lower levels of MBL and lower lectin pathway activation in the sera of BF +ve patients compared to BF –ve group, whereas the activation of classical and alternative pathways was not compromised. The frequency of patients with low or no MBL levels in the BF +ve group (31.3%) seems higher compared to those registered in the general population (>10%), revealing a possible connection between low MBL levels and brain fog onset. Thus, low levels of MBL not only reflect a reduced capacity to inhibit SARS-CoV-2 infection (31), but it can also predispose individuals to more severe symptoms during acute phase (65) as well as long COVID brain fog onset.

## 5 Conclusion

MBL level and lectin pathway activity are significantly reduced in subjects experiencing brain fog as a neuropsychiatric sequela in the post-acute phase of COVID-19. Thus, long COVID-associated brain fog can be listed among the variegate manifestations of increased susceptibility to infections and diseases induced by MBL deficiency.

## Data availability statement

The original contributions presented in the study are included in the article/supplementary material. Further inquiries can be directed to the corresponding authors.

## Ethics statement

The studies involving human participants were reviewed and approved by the local ethics committee (CEUR-2021-OS-48). The patients/participants provided their written informed consent to participate in this study.

## Author contributions

Conceptualization, GF, RB, CA and PM. Methodology MT, GF, AB and GM. Resources, GF, LR, ML, and PM. Data curation, CA, MT, AM, GM, and LR. Writing—original draft preparation, GF, CA, LR, AB, AM, M.L and PM. Writing—review and editing, RB, AB, UK, GF, LR and PM. supervision, RB and PM.
